# Effectiveness of implementing a preventive urinary catheter care bundle in hip fracture patients

**DOI:** 10.1177/17571774211060417

**Published:** 2022-02-15

**Authors:** Maria Frödin, Linda Ahlstrom, Brigid M. Gillespie, Cecilia Rogmark, Bengt Nellgård, Ewa Wikström, Annette Erichsen Andersson

**Affiliations:** 1 Institute of Health and Care Sciences, Sahlgrenska Academy, 174416 University of Gothenburg, Gothenburg, Sweden; 2 Department of Anesthesiology and Intensive Care Medicine, 70712 Sahlgrenska University Hospital, Gothenburg, Sweden; 3 Department of Orthopedics, Sahlgrenska University Hospital, Gothenburg, Sweden; 4 School of Nursing and Midwifery, 97562 Griffith University, Gold Coast, QLD, Australia; 5 Gold Coast University Hospital and Health Service, Southport, QLD, Australia; 6 Department of Orthopedics, Skane University Hospital, 59564 Lund University, Malmö, Sweden; 7 Swedish Hip Arthroplasty Register, Registercentrum VGR, Gothenburg, Sweden; 8 School of Business, Economics and Law, Department of Business Administration, 83210 University of Gothenburg, Gothenburg, Sweden

**Keywords:** Infection prevention, implementation, bundle intervention, acute hip fracture, catheter associated urinary tract infection

## Abstract

**Background:**

Urinary catheter (UC)–associated infections are one of the most common preventable healthcare-associated infections (HAIs) and they frequently occur in older, frail populations.

**Aim:**

The study aim was to describe the incidence of UC-associated infection in elderly patients undergoing hip fracture surgery after implementing a preventive care bundle.

**Methods:**

A longitudinal prospective study using a before-and-after design. The bundle was theory driven and involved the co-creation of a standard operational procedure, education and practical training sessions. Prospectively collected registry data were analysed. Univariable statistics and multivariable logistic regressions were used for analyses.

**Results:**

2,408 patients with an acute hip fracture were included into the study. There was an overall reduction in UC catheter associated-associated urinary tract infections, from 18.5% (*n* = 75/406) over time to 4.2% (*n* = 27/647). When adjusting for all identified confounders, patients in phase 4 were 74% less likely to contract an UC-associated infection (OR, 0.26; 95% CI, 0.15–0.45, *p* < 0.0001).

**Discussion:**

Bundled interventions can reduce UC-associated infections substantially, even in elderly frail patients. Partnership and co-creation as implementation strategies appear to be promising in the fight against HAI.

## Background

Urinary tract infections (UTIs) are one of the most common and preventable healthcare-associated infections (HAIs), the majority of which are related to urinary catheter (UC) use (Haque et al., 2018; Meddings et al., 2019; Umscheid et al., 2011). It is estimated that the risk of bacteriuria increases by 3–7% for each day the catheter is *in situ* (Lo et al., 2014). Generally, the side effects of UTI are less serious than those of other HAIs, but they may increase morbidity and mortality and contribute to the increased use of antibiotics (Haque et al., 2018; Suetens et al., 2018).

Patients undergoing acute hip fracture (AHF) surgery are exposed to several risk factors for contracting a UTI as they are elderly, mainly female, run an increased risk of urinary retention, are commonly admitted with bacteriuria and in need of UC (Bliemel et al., 2017; Carpintero et al., 2014; Johansson et al., 2002; Skelly et al., 1992). Indwelling urinary catheter (IUC) may be useful in AHF patients in the perioperative phase (Bliemel et al., 2017). However, using intermittent catheterisation (IC) might facilitate an early return to normal bladder function (Skelly et al., 1992). Nevertheless, avoiding complications such as UC-associated UTI is important as these frail patients have increased short- and long-term mortality compared with an age-matched population (Von Friesendorff et al., 2016).

Preventive strategies to reduce UC-associated infections are well described (Lo et al., 2014; Meddings et al., 2019). They consist of a bundle of measures, such as ensuring the education and training of healthcare workers (HCWs), an aseptic catheter insertion technique, avoiding unnecessary catheter placement, using an alternative to an IUC, short duration of the UC treatment, maintaining a closed UC system and avoiding urine backflow. Moreover, the best practice for peri-urethral cleansing has not yet been resolved (Lo et al., 2014; Fasugba et al., 2017; Meddings et al., 2019).

Furthermore, practice differences regarding catheterisation techniques exist, as well as misperceptions regarding the concept of *sterile*, *aseptic* and *clean* insertion techniques and the practical implications of using these approaches (Manojlovich et al., 2016; Kulbay and Tammelin, 2019; Vahr et al., 2013). Previous studies have indicated that healthcare workers have contradictory views on device-related best practice, that is, adhering to hand hygiene guidelines and aseptic techniques were not viewed by some as vital measures to prevent infection (Erichsen Andersson et al., 2018; Wikström et al., 2019). Moreover, other studies have reported low adherence to hand hygiene guidelines and aseptic techniques in relation to invasive procedures (Megeus et al., 2015a, 2015b; Allegranzi et al., 2017), contributing to the risk of device-related infections.

To summarise, the variability in UC management, deficits in knowledge and limited guideline implementation suggest a need for practice improvement among HCW. Likewise, the struggle to reduce avoidable HAIs, the rapid development of multidrug-resistant micro-organisms and the need to avoid adverse events in vulnerable groups suggest that finding an effective infection-prevention implementation strategy to minimise UC-associated infection is essential. The overall aim of this study was to describe changes in the incidence of UC-associated infections following the implementation of an infection-prevention bundle, aimed at elderly patients undergoing AHF surgery.

## Method

### Study design

This 4-phase, single-centre implementation study used a longitudinal prospective before-and-after design, between mid-2015 and mid-2019. The main outcome parameter over time was the number of UC-associated infections among patients after AHF surgery, while implementing an infection-prevention bundle intervention.

### Setting

This study was set at an orthopaedic centre at a university hospital in Sweden, performing AHF surgery on approximately 900 patients annually.

At the study hospital, the infection-prevention measures for AHF patients consisted of ≥1 preoperative antiseptic double shower and prophylactic antibiotics given intravenously, 2 g of cloxacillin, within 45–30 min prior to surgery. Internal fixation with surgery ≥2 h necessitated a second antibiotic dose. For arthroplasty, another two doses were administered 2 and 6 h after the initial dose. A dose of 600 mg of clindamycin was administered to patients with penicillin allergy, with a second dose 4 h after the first dose for arthroplasty. The hospital strategy for reducing UC-associated infections before the intervention was to use the *clean* intermittent catheterisation insertion technique, that is, using non-sterile gloves and forceps, with a pre-wash with soap and water (or no wash at all for IC), if patients had urinary retention or post-void residual urine of ≥400 mL. If the patient’s medical status required an IUC, a physician’s order was requested. The prompt removal of the catheter on the day after surgery, after mobilisation to an upright position, was routine, if no indication for the continued use of an IUC was present. A visual tool (a dwelling catheter sign magnet on the patient board at the nurses’ desks) was used as a reminder of the patients who had an IUC *in situ* to ensure removal when indications were no longer present.

### The intervention

In 2015, the Safe Hands study (Clinical Trials. gov ID: NCT02983136) developed, tested and implemented a programme for improving hand hygiene and aseptic techniques in the operating room department (OD) (Erichsen Andersson et al., 2018; Wikström et al., 2019). The programme strategy was linked to theories on organisational learning, culture, change and dialogue (Isaacs, 2002; Schein, 2010) and entailed leadership support and the facilitation of interprofessional dialogue and co-creation (Bason, 2018). A description of the programme development has been published (Erichsen Andersson et al., 2018). The UC management was identified as one of the most urgent procedures to further develop and modify, due to practice variability. The core components in phase 1 of the intervention consisted of the development of standard operating procedures (SOP) for device-related, infection-prone procedures, through an iterative and co-creative process involving managers, registered nurses (RNs), physicians and nurse assistants in the OD. From this initial intervention, a bundle comprising an SOP for UC insertion, an educational programme, training sessions and a skills test was developed in phase 2. In phases 3 and 4, the bundle was implemented in the units involved in the care of AHF patients, that is, the emergency room, OD the ortho-geriatric wards and the intensive care and post-anaesthetic care unit. The participating HCWs were RNs and nurse assistants working at the units (see Supplementary Box S1 for an overview of the implementation process and the bundled components and Supplementary Box S2 for the timeframe of the implementation (supplementary material)).

### Data collection

Patient-related data were extracted from the local quality register of patients undergoing AHF surgery at the study site, from June 2015 to April 2019. AHF patients, ≥65 years of age, admitted to one of the ortho-geriatric wards and included in the local quality registry were included. Patients residing outside Sweden, previously included in the study due to contralateral AHF surgery, length of stay in hospital (HLOS) ≤2 days, resection arthroplasty (Girdlestone procedure), IUC *in situ* before admission, chronic UC, suprapubic catheter, urostomy, intermittent self-catheterisation, on dialysis treatment and not catheterised during their hospital stay were excluded (see Supplementary Figure S1 (supplementary material)).

Data extracted for study purposes were scrutinised against the medical records by an experienced RN, specialising in infection control and anaesthetic care. Variables derived from the registry were age (years), gender (female/male), ASA classification score (I-IV) (ASA House of Delegates/Executive Committee, 2014), diabetes (yes/no), HLOS (days), UTI and UC-associated infections. Data on catheterisation treatment were also extracted when the patient was either treated only with an indwelling catheter, or via intermittent catheterisation or a combination of both. We further noted the location of catheter insertion (emergency room, ortho-geriatric ward, OD post-anaesthesia care unit/intensive care unit or >1 location), number of reinsertions, number of intermittent catheterisations and, finally, the number of catheter days.

### Definition of urinary catheter–associated infections

The patients were registered as having a UC-associated infection if a physician had made the diagnosis and prescribed antimicrobial treatment during the hospital stay. The UTI had to occur >2 days after admission to the hospital, admission day defined as day 1, related to catheterisation treatment in line with the European Centre for Disease Prevention and Control definition for point prevalence survey (European Centre for Disease Prevention and Control, 2016). The routine was to send a urine specimen for analysis if the patient presented symptoms of a UTI. Only two patients in this cohort received antimicrobial treatment for UTI where no urine specimens were analysed, with a clear symptom effect. Patients on antimicrobial treatment for UTI on admission were not defined as having a UC-associated infection, unless a new culture showed a new microbe that required treatment. Similarly, patients with asymptomatic bacteriuria were not considered to have a UC-associated infection.

### Statistical analysis

Categorical variables are presented as numbers (%) and continuous variables as the mean (SD, min-max). The overall percentage of UC-associated infections was compared between phases and over the phases of the intervention and it was then stratified into groups according to catheterisation treatment; IUC, IC and IUC+IC. The Mantel–Haenszel chi-square test was used for ordered categorical variables, while the Jonckheere–Terpstra test was used for continuous variables analysed over ordered groups. For pairwise comparisons between groups, Fisher’s exact test (2-sided) was used for dichotomous variables, while Fisher’s non-parametric permutation test was used for continuous variables.

For the identification of factors related to UC-associated infections, univariable logistic regression analysis was performed (presented as the odds ratio (OR) and 95% confidence interval (CI)). Phase 1 was used as a reference for tests against phases 2–4. All *p*-values were 2-sided and conducted at the 5% significance level. The area under the ROC curve (AUC) was calculated to describe goodness of fit (Hosmer et al., 2013). Multivariable logistic regression was used to analyse the effect of phase 4 versus phase 1 on UC-associated infections, with adjustments for the confounders of age, gender, ASA, HLOS, IUC days, IUC reinsertion and number of IC. The adjusted odds ratio with 95% CI was calculated and the area under the ROC curve was calculated to describe model fit. SAS version 9.4 was used for all statistical analyses.

A joinpoint regression analysis was constructed to find breaking points in UC-infection trends during the time periods and to estimate the quarterly change in UC-associated infections with a 95% confidence interval.

### Ethics

Ethical approval was obtained from the Regional Ethical Review Board in Gothenburg, Sweden (reference number 166–15 and 327–17). The hospital’s Chief Executive Officer and the departmental managers approved the study. Patients received written information about the quality registry, information on who to contact about their registry data and if they wanted to withdraw their participation, or if they did not want their data to be used for research purposes. No formal written consent is required when using registry data. The study was conducted in accordance with the Helsinki Declaration (World Medical Association, 2014).

## Results

A total of 2408 patients with AHF were included, following the exclusion of 502 patients (see Supplementary Figure S1 (supplementary material)). The patients’ mean age was 84 years (range 65–102 years) and two-thirds were female. There were no significant differences between the patient demographics in the different phases (1–4) in terms of age, gender, ASA score and diabetes (Table 1). For HLOS, there was a significant difference between groups over the phases; 14.7 days in phase 1 vs 10.5 days in phase 4 (*p* < 0.0001).

**Table 1.** Patient demographics and clinical data for patients with an acute hip fracture (*N*=2408), phases 1 to 4.

Characteristic	Phase 1, *n* = 406	Phase 2, *n* = 655	Phase 3, *n* = 700	Phase 4, *n* = 647	*p-*value for trend
Age (years)	83.8 (8.0),	84.8 (7.7),	83.9 (8.3),	83.8 (8.3),	0.30^±^
	65–102	65–102	65–101	65–102	
Female gender	294 (72.4)	446 (68.1)	493 (70.4)	469 (72.5)	0.53^+^
ASA classification score					
I	15 (3.7)	14 (2.1)	11 (1.6)	28 (4.3)	
II	165 (40.6)	264 (40.6)	257 (36.7)	268 (41.4)	
III	196 (48.3)	333 (50.8)	390 (55.7)	322 (49.8)	
IV	30 (7.4)	44 (6.7)	42 (6.0)	29 (4.5)	0.24^+^
Diabetes	57 (14.0)	105 (16.0)	102 (14.6)	103 (15.9)	0.63^+^
Hospital length of stay (days)	14.7 (7.2),	13.4 (7.7),	12.2 (6.4),	10.5 (5.2),	<0.0001^±^*
	3–52	3–68	3–55	3–46	

Values are given as the mean and standard deviation (SD), min–max for continuous variables and n (%) for categorical variables. ^+^ Mantel–Haenszel chi-square test and ^±^ Jonckheere–Terpstra test for continuous variables. Values of p<0.05 were considered statistically significant. ^*^ *p*-value <0.05.

We identified an overall significant decrease in the total numbers of patients with UC-associated infections from 18.5% (*n* = 75/406) in phase 1 vs 4.2% in phase 4 (*n* = 27/647), (*p* < 0.0001). In the IUC-treatment group, there was a reduction in UC-associated infections between the phases; phase 1, 14.9%, (*n* = 17/114), *v*s 3.1% (*n* = 15/490), in phase 4, (*p* < 0.0001). Similar patterns were seen for the IUC+IC and IC groups. For detailed results on UC-associated infections and related variables, see Table 2. A significant difference in UC treatment between the phases was identified (*p* < 0.0001). More patients received an IUC; phase 1, 28.1% (*n* = 114/406) vs phase 4, 75.7% (*n* = 490/647) and fewer patients also had both IUC+IC, phase 1, 35% (*n* = 142/406) vs phase 4, 20.7% (*n* = 134/647) and IC, phase 1, 36.9% (*n* = 150/406) vs 3.6% (*n* = 23/647) (see Supplementary Table S3 for information on the location of catheter placement from phase 1 to 4, supplementary).

**Table 2.** Urinary catheter–associated infection and related data stratified by catheterisation treatment among patients with an acute hip fracture in the different phases of the intervention.

Method	Variables	Phase 1	Phase 2	Phase 3	Phase 4	*p*-value
						Phase 1 vs. phase 4
Indwelling urinary catheter		*n*=114	*n*=229	*n*=396	*n*=490	
*IUC*	UC-associated infection	17 (14.9)	19 (8.3)	23 (5.8)	15 (3.1)	<0.0001^**±**^
	IUC days	3.83 (3.66), 0.5–20	3.40 (3.74), 0.5–30	4.25 (4.65), 1–48	3.45 (2.44), 0.5–20	0.18^+^
	IUC reinsertion	3 (2.6)	9 (3.9)	31 (7.8)	24 (4.9)	0.43^**±**^
Indwelling urinary catheter and intermittent catheterisation		*n* = 142	*n* = 270	*n* = 228	*n* = 134	
*IUC+IC*	UC-associated infection	37 (26.1)	39 (14.4)	25 (11.0)	11 (8.2)	0.0001^**±**^
	IUC days	4.43 (4.16)	5.23 (4.86)	6.07 (5.78)	5.14 (4.15)	0.16^+^
		0.5–21	1–26	1–31	1–21	
	Number of IC	3.80 (4.08),	3.09 (2.93),	2.17 (1.67), 1–10	1.81 (1.11),	<0.0001^+^
		1–25	1–16		1–7	
	IUC reinsertion	28 (19.7)	69 (25.6)	79 (34.6)	44 (32.8)	0.019^**±**^
Intermittent catheterisation		*n*=150	*n*=156	*n*=76	*n*=23	
*IC*	UC-associated infection	21 (14.0)	24 (15.4)	2 (2.6) *	1 (4.3)	0.34^**±**^
	Number of IC	2.46 (1.72),	2.26 (1.70),	1.79 (1.26), 1–7	1.17 (0.49),	<0.0001^+^
		1–10	1–8		1–3	

Categorical variables are presented as n(%), continuous as the mean, standard deviation (SD) and min-max. IUC days include reinsertion days. ^**±**^ Fisher’s exact test (2-sided) was used for comparisons between groups for dichotomous variables and ± Fisher’s non-parametric permutation test was used for continuous variables. *p*-values of < 0.05 were considered statistically significant. * *p*-value 0.008, phase 1 vs. phase 3.

The odds of contracting a UC-associated infection were reduced by 42%, over the phases (OR 0.58, 95% CI, 0.50–0.66, *p* < 0.0001). The odds of contracting a UC-associated infection were 2.1 (95% CI 1.60–2.75, *p* < 0.0001) times higher in the IUC+IC group and the odds were 41% lower (95% CI 0.31–0.54, *p* < 0.0001) for patients with IUC (see Supplementary Table S4 for the univariate regression results, supplementary).

In the model building approach, the independent variables that were significantly associated with the dependent variable, as well as previously known prognostic risk factors, were entered simultaneously into the model. The significant variables that were entered were time of intervention (phases 1–4), IUC days, reinsertion of the UC, number of IC, HLOS and previously known prognostic risk factors, that is, age, gender (female) and ASA classification scores. The multivariable model indicated that the odds of contracting a UC-associated infection in phase 1 were 4 times higher vs patients in phase 4 (adjusted OR 0.26; 95% CI, 0.15–0.45, *p* < 0.0001) (Table 3).

**Table 3.** Multivariable logistic regression analysis.

Variables	OR* (95% CI)	*p-*value	OR (95% CI)	*p-*value	Area under ROC curve
Numbers of UC-associated infections	Unadjusted		Adjusted for confounders*		
Yes= 102, No=951					
Time of intervention,	0.19 (0.12–0.30)	<0.0001	0.26 (0.15–0.45)	<0.0001	0.77
Phase 4 versus phase 1					

CI, confidence interval; OR, odds ratio. * Confounders; age, gender, ASA, HLOS, IUC days, IUC reinsertion, number of IC.

From the joinpoint regression model, the quarterly percentage change (QPC) was estimated to be a 12% reduction in infection prevalence with 95% CI (−15.5; −8.3), from Q2 2015 to Q1 2019. The QPC differed significantly from zero (alpha level <0.05) (Supplementary Figure S2).

## Discussion

This paper describes the efficacy of implementing an infection-prevention bundle, aimed at elderly patients undergoing AHF surgery. We observed a significant trajectory reduction in UC-associated infections during the implementation period. Our results are in line with the knowledge that UC-associated infections are largely preventable (Umscheid et al., 2011) and can also be found in other multifaceted implementation studies (Stéphan et al., 2006; Saint et al., 2016). The reduction occurred despite an increase in the proportion of patients with IUC. Further, more patients received an IUC in the ER than in the OD in phase 4 vs phase 1. This is in contrast to a study which found that an IUC inserted outside the OD was a risk factor for infections (Barbadoro et al., 2015). Reinforced awareness of appropriate indications for IUC in this group of patients might explain this outcome.

We also found that IUC days, reinsertion of an IUC and number of IC were associated with an increased risk of UTI. This might be explained by the risk of introducing micro-organisms into the bladder during catheter insertion. Bacterial growth extraluminally of the catheter has been identified on day 1, while intraluminal growth has been identified on day 4 (Barford et al., 2008), similar to a pilot study identifying an increasing number of colony-forming units extraluminally of the catheter, from the bladder and out (Foxman et al., 2012). The hypothesis is that the contamination of the outside of the catheter occurred during insertion, from the peri-urethral area. Based on clinical experience, it is easy to contaminate the catheter, especially the catheter tip, before insertion.

Further, intermittent catheterisation is also a risk factor for UTI and both too few IC (causing excessive urine volume) and inadequate emptying (residual volume) increases the risk of UTI, as stagnation of urine promotes bacteria growth (Newman and Willson, 2011; Wyndaele, 2002).

For the IC-treatment group, the reduction was found during phase 3. This may be explained by the fact that in phases 1 and 2, the RNs and nurse assistants expressed strong doubt about the necessity of using aseptic techniques and peri-urethral cleansing with chlorhexidine gluconate (CHX) during IC, due to the ‘quick in and out’ procedure. In phases 3 and 4, we noted greater acceptance of the new practice and the significant reduction in phase 3 shows the potential efficacy of our care bundle in the IC group.

The reported incidence of UC-associated infections at baseline is within the upper range of reported incidents of antimicrobial treatment for symptomatic UTI in this patient group (it varies from one 10th to one quarter of the patients) (Bliemel et al., 2017; Hälleberg Nyman et al., 2011; Hedström et al., 1999; Kamel, 2005). The variability in reported incidence has several explanations, one of which is the use of different definitions of UC-associated infections.

Further, diabetes did not predict infection in our population, similar to a previous study (Bliemel et al., 2017) and contrary to another study (Hälleberg Nyman et al., 2011).

### The intervention

The care bundle includes several preventive measures that are included in the preventive recommendations, apart from the peri-urethral cleansing (Lo et al., 2014). A recent meta-analysis failed to identify any clear advantages when using CHX for peri-urethral cleansing (Fasugba et al., 2017). On the other hand, Fasugba et al. (2019) performed a step-wise multicentre randomised controlled trial to assess the efficacy of CHX solution 0.1% vs sodium chloride 0.9%, as cleansing before catheterisation. They found a large reduction in both UC-associated infection incidence and asymptomatic bacteriuria. As our intervention comprised a bundle of actions, it is difficult to single out the effect of CHX use. However, we agree with Fasugba and co-authors that CHX may be important in UC-prevention strategies (Mitchell et al., 2019), not least its ability to eradicate multidrug-resistant micro-organism biofilm formation (Günther et al., 2020), but the question merits further investigation.

Our implementation strategies were based on organisational learning and culture theories, co-creation, partnership and dialogue between leaders, RNs, physicians and experts in infection prevention (Erichsen Andersson et al., 2018). The concept of partnership implies that we meet as equals and with mutual respect. Within the simulation training, the intention was to create a safe place for mutual learning, free of blame and shame, and to nudge technical skills under guidance. This atmosphere encouraged questioning and seeing mistakes and failures as a natural part of learning. As a result, the implementation strategy appears to be promising when it comes to creating a collective action and normalising routines to improve hand hygiene and aseptic techniques in preventable infection-prone invasive procedures (May, 2013).

### Strengths and limitations

This study has limitations. As it is a single-centre study based on registry data, it has inherent weaknesses. The register does not collect information on all potential confounders that may affect outcome. Our results must therefore be interpreted with caution. Furthermore, temporal trends and organisational changes in the healthcare system are all factors that may have influenced outcome.

To enhance study rigour, we used a robust data set and took important steps to validate the registry data by meticulously checking them against medical, nursing and laboratory records. We have used statistical tests to control for potential confounders to identify other variables affecting the outcome. In our data, HLOS was reduced over the phases, which could have been an effect of our intervention, as an infection reduction may shorten HLOS (Bliemel et al., 2017). However, HLOS as an outcome variable is difficult to interpret, as it can be affected by other factors such as adverse events and planning for further care. Nevertheless, the lack of available infection data after hospital discharge is problematic and limits our ability to detect infections after discharge. Moreover, using a UTI definition based on physician prescription of antimicrobial agents means that we might have identified incorrectly diagnosed infections. However, the study hospital adhered to the national collaboration against the overuse of antimicrobial agents, and as a result, asymptomatic bacteriuria was not screened for or treated. Further, there was close collaboration with consultant specialists in infection prevention to ensure that the antimicrobial agent had the smallest spectrum for the identified micro-organisms.

We did not expect the low proportion of completed certificates. We aimed for least >75% of nursing staff completing the certificate after 5 months. However, during the intervention, the participants were encouraged to use the new practice in their daily work for all patients in need of UC and they had their ward-specific facilitators. So, the level of completed certificates might not reflect the actual level of implementation. The fact that we did not measure adherence to the SOP in clinical practice is nonetheless a weakness.

## Conclusion

Urinary catheter–associated infection was significantly reduced by the systematic implementation of a bundled intervention in elderly patients undergoing acute hip fracture surgery. Ensuring an adequate level of knowledge, competence and practical skills among the nursing staff is essential to reduce UC-associated infections.

## Supplemental Material

sj-pdf-1-bji-10.1177_17571774211060417 – Supplemental Material for Effectiveness of implementing a preventive urinary catheter care bundle in hip fracture patients Click here for additional data file. Supplemental Material, sj-pdf-1-bji-10.1177_17571774211060417 for Effectiveness of implementing a preventive urinary catheter care bundle in hip fracture patients by Maria Frödin, Linda Ahlstrom, Brigid M. Gillespie, Cecilia Rogmark, Bengt Nellgård, Ewa Wikström and Annette Erichsen Andersson in Journal of Infection Prevention

sj-pdf-2-bji-10.1177_17571774211060417 – Supplemental Material for Effectiveness of implementing a preventive urinary catheter care bundle in hip fracture patients Click here for additional data file. Supplemental Material, sj-pdf-2-bji-10.1177_17571774211060417 for Effectiveness of implementing a preventive urinary catheter care bundle in hip fracture patients by Maria Frödin, Linda Ahlstrom, Brigid M. Gillespie, Cecilia Rogmark, Bengt Nellgård, Ewa Wikström and Annette Erichsen Andersson in Journal of Infection Prevention

sj-pdf-3-bji-10.1177_17571774211060417 – Supplemental Material for Effectiveness of implementing a preventive urinary catheter care bundle in hip fracture patients Click here for additional data file. Supplemental Material, sj-pdf-3-bji-10.1177_17571774211060417 for Effectiveness of implementing a preventive urinary catheter care bundle in hip fracture patients by Maria Frödin, Linda Ahlstrom, Brigid M. Gillespie, Cecilia Rogmark, Bengt Nellgård, Ewa Wikström and Annette Erichsen Andersson in Journal of Infection Prevention

sj-pdf-4-bji-10.1177_17571774211060417 – Supplemental Material for Effectiveness of implementing a preventive urinary catheter care bundle in hip fracture patients Click here for additional data file. Supplemental Material, sj-pdf-4-bji-10.1177_17571774211060417 for Effectiveness of implementing a preventive urinary catheter care bundle in hip fracture patients by Maria Frödin, Linda Ahlstrom, Brigid M. Gillespie, Cecilia Rogmark, Bengt Nellgård, Ewa Wikström and Annette Erichsen Andersson in Journal of Infection Prevention

sj-pdf-5-bji-10.1177_17571774211060417 – Supplemental Material for Effectiveness of implementing a preventive urinary catheter care bundle in hip fracture patients Click here for additional data file. Supplemental Material, sj-pdf-5-bji-10.1177_17571774211060417 for Effectiveness of implementing a preventive urinary catheter care bundle in hip fracture patients by Maria Frödin, Linda Ahlstrom, Brigid M. Gillespie, Cecilia Rogmark, Bengt Nellgård, Ewa Wikström and Annette Erichsen Andersson in Journal of Infection Prevention

## References

[bibr1-17571774211060417] Allegranzi B Stewardson AJ Pittet D. (2017) Compliance with hand hygiene best practices. In: Pittet D Boyce JM Allegranzi B (eds) Hand Hygiene: A Handbook for Medical Professionals. Chichester: Wiley Blackwell, pp. 76–84.

[bibr2-17571774211060417] ASA House of Delegates/Executive Committee (2014) ASA physical status classification system. https://www.asahq.org/standards-and-guidelines/asa-physical-status-classification-system.

[bibr3-17571774211060417] Barbadoro P Labricciosa FM Recanatini C , et al. (2015) Catheter-associated urinary tract infection: Role of the setting of catheter insertion. Am J Infect Control 43: 707–710. 25840715 10.1016/j.ajic.2015.02.011

[bibr4-17571774211060417] Barford JMT Anson K Hu Y , et al. (2008) A model of catheter-associated urinary tract infection initiated by bacterial contamination of the catheter tip. BJU Int 102: 67–74. 18284413 10.1111/j.1464-410X.2008.07465.x

[bibr5-17571774211060417] Bason C (2018) Leading Public Sector Innovation 2E: Co-creating for a Better Society. Bristol, UK: Policy Press.

[bibr6-17571774211060417] Bliemel C Buecking B Hack J , et al. (2017) Urinary tract infection in patients with hip fracture: An underestimated event? Geriatr Gerontol Int 17: 2369–2375. 28621029 10.1111/ggi.13077

[bibr7-17571774211060417] Carpintero P Caeiro JR Carpintero R , et al. (2014) Complications of hip fractures: A review. World J Orthop 5: 402. 25232517 10.5312/wjo.v5.i4.402 PMC4133447

[bibr8-17571774211060417] Erichsen Andersson A Frödin M Dellenborg L , et al. (2018) Iterative co-creation for improved hand hygiene and aseptic techniques in the operating room: experiences from the safe hands study. BMC Health Serv Res 18: 2. 29301519 10.1186/s12913-017-2783-1 PMC5753493

[bibr9-17571774211060417] European Centre for Disease Prevention and Control (2016) Point prevalence survey of healthcare-associated infections and antimicrobial use in European acute care hospitals. https://www.ecdc.europa.eu/sites/default/files/media/en/publications/Publications/PPS-HAI-antimicrobial-use-EU-acute-care-hospitals-V5-3.pdf.

[bibr10-17571774211060417] Fasugba O Cheng AC Gregory V , et al. (2019) Chlorhexidine for meatal cleaning in reducing catheter-associated urinary tract infections: a multicentre stepped-wedge randomised controlled trial. Lancet Infect Dis 19: 611–619. 30987814 10.1016/S1473-3099(18)30736-9

[bibr11-17571774211060417] Fasugba O Koerner J Mitchell BG , et al. (2017) Systematic review and meta-analysis of the effectiveness of antiseptic agents for meatal cleaning in the prevention of catheter-associated urinary tract infections. J Hosp Infec 95: 233–242. 27986361 10.1016/j.jhin.2016.10.025

[bibr12-17571774211060417] Foxman B Wu J Farrer EC , et al. (2012) Early development of bacterial community diversity in emergently placed urinary catheters. BMC Res Note 5: 332. 10.1186/1756-0500-5-332 PMC3500218 22738659

[bibr13-17571774211060417] Günther F Blessing B Dapunt U , et al. (2020) Ability of chlorhexidine, octenidine, polyhexanide and chloroxylenol to inhibit metabolism of biofilm-forming clinical multidrug-resistant organisms. J Infec Prev 22: 12–18. 33841557 10.1177/1757177420963829 PMC7841706

[bibr14-17571774211060417] Haque M Sartelli M McKimm J , et al. (2018) Health care-associated infections—an overview. Infect Drug Resis 11: 2321–2333. 10.2147/IDR.S177247 PMC6245375 30532565

[bibr15-17571774211060417] Hedström M Gröndal L Ahl T (1999) Urinary tract infection in patients with hip fractures. Injury 30: 341–343. 10505128 10.1016/s0020-1383(99)00094-7

[bibr16-17571774211060417] Hosmer DW Jr Lemeshow S Sturdivant RX (2013) Applied Logistic Regression. New York, NY: John Wiley & Sons.

[bibr17-17571774211060417] Hälleberg Nyman M Johansson J-E Persson K , et al. (2011) A prospective study of nosocomial urinary tract infection in hip fracture patients: Nosocomial UTI in hip fracture patients. J Clin Nurs 20: 2531–2539. 21733026 10.1111/j.1365-2702.2011.03769.x

[bibr18-17571774211060417] Isaacs WN. (2002) Creating a shared field of meaning: An action theory of dialogue. In: Roberts NC (ed) The Transformative Power of Dialogue. Bingley: Emerald Group Publishing Limited, pp. 203–241.

[bibr19-17571774211060417] Johansson I Athlin E Frykholm L , et al. (2002) Intermittent versus indwelling catheters for older patients with hip fractures. J Clin Nurs 11: 651–656. 12201892 10.1046/j.1365-2702.2002.00646.x

[bibr20-17571774211060417] Kamel HK. (2005) The frequency and factors linked to a urinary tract infection coding in patients undergoing hip fracture surgery. J Am Med Dir Assoc 6: 316–320. 16165072 10.1016/j.jamda.2005.04.005

[bibr21-17571774211060417] Kulbay A Tammelin A (2019) Clean or sterile technique when inserting indwelling urinary catheter: An evaluation of nurses’ and assistant nurses’ interpretations of a guideline at an acute-care hospital in Sweden. Nord J Nurs Res 39: 92–97.

[bibr22-17571774211060417] Lo E Nicolle LE Coffin SE , et al. (2014) Strategies to prevent catheter-associated urinary tract infections in acute care hospitals: 2014 update. Infect Control Hosp Epidemiol 35: S32–S47. 25376068

[bibr23-17571774211060417] Manojlovich M Saint S Meddings J , et al. (2016) Indwelling urinary catheter insertion practices in the emergency department: an observational study. Infect Control Hosp Epidemiol 37: 117–119. 26434781 10.1017/ice.2015.238

[bibr24-17571774211060417] May C (2013) Towards a general theory of implementation. Implement Sci 8: 18. 23406398 10.1186/1748-5908-8-18 PMC3602092

[bibr25-17571774211060417] Meddings J Manojlovich M Fowler KE , et al. (2019) A tiered approach for preventing catheter-associated urinary tract infection. Ann Int Med 171: S30–S37. 31569226 10.7326/M18-3471

[bibr26-17571774211060417] Megeus V Nilsson K Karlsson J , et al. (2015a) Hand hygiene and aseptic techniques during routine anesthetic care—observations in the operating room. Antimicrob Resist Infect Control 4: 5. 25685334 10.1186/s13756-015-0042-y PMC4328079

[bibr27-17571774211060417] Megeus V Nilsson K Karlsson J , et al. (2015b) Hand contamination, cross-transmission, and risk-associated behaviors: an observational study of team members in ORs. AORN J 102: 645.e1–645.e12. 26616330 10.1016/j.aorn.2015.06.018

[bibr28-17571774211060417] Mitchell BG Cheng AC Fasugba O , et al. (2019) Chlorhexidine for prevention of catheter-associated urinary tract infections: the totality of evidence—Authors’ reply. Lancet Infect Dis 19: 808–809. 10.1016/S1473-3099(19)30349-4 31345452

[bibr29-17571774211060417] Newman DK Willson MM (2011) Review of intermittent catheterization and current best practices. Urol Nurs 31: 12–28. 21542441

[bibr30-17571774211060417] Saint S Greene MT Krein SL , et al. (2016) A program to prevent catheter-associated urinary tract infection in acute care. NEJM 374: 2111–2119. 27248619 10.1056/NEJMoa1504906 PMC9661888

[bibr31-17571774211060417] Schein E. (2010) Organizational Culture and Leadership. New York, NY: Wiley.

[bibr32-17571774211060417] Skelly J Guyatt G Kalbfleisch R , et al. (1992) Management of urinary retention after surgical repair of hip fracture. CMAJ 146: 1185. 1555145 PMC1488328

[bibr33-17571774211060417] Stéphan F Sax H Wachsmuth M , et al. (2006) Reduction of urinary tract infection and antibiotic use after surgery: a controlled, prospective, before-after intervention study. Clin Infect Dis 42: 1544–1551. 16652311 10.1086/503837

[bibr34-17571774211060417] Suetens C Latour K Kärki T , et al. (2018) Prevalence of healthcare-associated infections, estimated incidence and composite antimicrobial resistance index in acute care hospitals and long-term care facilities: results from two European point prevalence surveys, 2016 to 2017. Euro Surveill 23: 1800516. 10.2807/1560-7917.ES.2018.23.46.1800516 PMC6247459 30458912

[bibr35-17571774211060417] Umscheid CA Rajender Agarwal MD M Kendal Williams MDM , et al. (2011) Estimating the proportion of healthcare-associated infections that are reasonably preventable and the related mortality and costs. Infect Control Hosp Epidemiol 32: 101–114. 21460463 10.1086/657912

[bibr36-17571774211060417] Vahr S Cobussen-Boekhorst H Eikenboom J , et al. (2013) Evidence-based guidelines for best practice in urological health care catheterisation. Urethral Intermittent in Adults. https://nurses.uroweb.org/wp-content/uploads/2013_EAUN_Guideline_Milan_2013-Lr_DEF.pdf.

[bibr37-17571774211060417] von Friesendorff M McGuigan FE Wizert A , et al. (2016) Hip fracture, mortality risk, and cause of death over two decades. Osteopor Int 27: 2945–2953. 10.1007/s00198-016-3616-5 27172936

[bibr38-17571774211060417] Wikström E Dellenborg L Wallin L , et al. (2019) The safe hands study: implementing aseptic techniques in the operating room: facilitating mechanisms for contextual negotiation and collective action. Am J Infect Control 47: 251–257. 30449454 10.1016/j.ajic.2018.08.024

[bibr39-17571774211060417] World Medical Association (2014) World medical association declaration of Helsinki: ethical principles for medical research involving human subjects. J Am College Dent 81: 14–18. 25951678

[bibr40-17571774211060417] Wyndaele JJ. (2002) Complications of intermittent catheterization: their prevention and treatment. Spinal Cord 40: 536–541. 12235537 10.1038/sj.sc.3101348

