# Supplementary material for: Effectiveness of implementing a preventive urinary catheter care bundle in hip fracture patients
Source: J Infect Prev. 2022 Feb 15;23(2):41–8. doi: 10.1177/17571774211060417 (PMC8941588; doi:10.1177/17571774211060417)
Supplement: sj-pdf-1-bji-10.1177_17571774211060417 – Supplemental Material for Effectiveness of implementing a preventive urinary catheter care bundle in hip fracture patients [file sj-pdf-1-bji-10.1177_17571774211060417.pdf]

Box 1. Description of the implementation process and the components of the bundle

Brief description of the implementation process

Phase 1. The implementation of an infection prevention bundle (hand hygiene, aseptic techniques and postoperative infections) in the operating room (OR), built on partnership, dialogue and co-creation. Co-creating standard operating procedures (SOP) for device-related infection-prone procedures (Erichsen Andersson et al., 2018; Wikström et al., 2018).

Phase 2. An action plan for implementing the care bundle was developed with the key stakeholders involved in the care of patients undergoing hip fracture surgery. To ensure the sustainability of the intervention, each unit's ward manager appointed two specially trained nurses (or one nurse and one nurse assistant) to facilitate the introduction of the bundle at their unit. These expert nurses created a network group for the further implementation process in the patient pathway of these patients, as part of the co-creation process.

Phase 3. The learning activity and educational tool was set as a mandatory task.

Phase 4. The wards using and taking ownership of the bundle. Post measurement of patient outcome.

The components of the bundle

The bundle consisted of 1) a standard operational procedure, 2) learning activities and educational tools and 3) continued use of the IUC magnet as a reminder.

*The **standard operational procedure*** gave detailed step-by-step instructions for the catheterisation procedure and included the following elements:

- aseptic preparation of all necessary material,
- pre-wash of the peri-urethral area with a 4% chlorhexidine gluconate impregnated sponge,
- use of sterile gloves and a sterile equipment set,
- a peer to facilitate aseptic techniques and to ensure the sterility of the catheter until inserted,
- a closed urinary catheter system, ensuring free drainage of the urine and avoiding backflow,
- a careful fixation technique to avoid traction of the catheter.

Learning activities and educational tools

The learning activities and the educational tools were co-created by two certified OR nurses, a nurse assistant, a clinical instructor and two of the researchers. The participating wards received ***a state-of- the-art lecture*** on infection prevention, hand hygiene and aseptic techniques, change management and a presentation of the current *data on UC-associated infections*. ***An instruction film*** was available to all staff via an electronic learning platform. The film included a thorough description of the SOP on how to insert an indwelling catheter or intermittent catheterisation in men and women. ***A computerised quiz*** was launched, with questions regarding appropriate indications for urinary catheterisation, its risks, benefits and bladder distension prevention. No limitation on the number of attempts needed to pass the test. ***Peer-to-peer clinical skills training*** was undertaken by a specially educated nurse, nurse assistant or one of the researchers. The clinical skills test had a flexible time frame (15-45 minutes) and functioned as a learning opportunity to "nudge" the practical skills of participants to manage all the steps in aseptic catheterisation. After completing the learning activities, participants received a UC certificate. The result was registered on the learning platform and was available to both the participants and the managers for follow-up.

Box 2. Time frame of the implementation process

Phases of the intervention	Time periods n=months	Intervention actions	Activity taking place
**1**	13	Implementing an infection prevention innovation in the OR	Co-creation of SOP to improve hand hygiene and aseptic techniques during invasive procedures in the OR. The UC SOP was set as a new electronic guideline in the OR.
**2**	11	Consensus decision to further disseminate the SOP in the hip-fracture patients' care pathway, via the development of a UC bundle	1. Co-creating the instruction film, a quiz and a simulation test for the UC certificate on the electronic learning platform<br>2. Preplanning and disseminating the bundle in the hip-fracture patient's care pathway; ER, PACU/ICU, OR and three ortho-geriatric wards<br>3. Educating expert nurses and nurse assistants in infection prevention and the UC bundle. These nurses facilitated the implementation process.
**3**	12	Implementing the UC certificate in the hip-fracture patients' care pathway	All the involved units received a state-of-the-art lecture on infection prevention, the bundle strategy for UTI prevention. In January 2018, the electronic UC certificate was implemented. The units had five months to complete the certificate.*
**4**	11	Post measurements	The units continued to use the learning platform and educate newly employed staff in the UC certificate, taking over ownership.

**Percentage of staff with a completed electronic UC certificate on the different wards: emergency room, ER, 58% (n=86), operating room, OR, 51% (n=111), ortho-geriatric wards, OGW:1, 34% (n=44), OGW:2, 53% (n=41), OGW:3, 53% (n=50) and post-anaesthesia care unit, PACU/intensive care unit/intensive care unit, ICU, 76% (n=69). Total completed certificates after five months 56% (n=223 of 401). The number includes staff working part time and on sick leave. Due to staff turnover, the OGW were reduced from three to two wards at the beginning of phase 4.*

UC = urinary catheter, UTI = urinary tract infection, SOP = standard operating procedure

Erichsen Andersson A, Frödin M, Dellenborg L, et al. (2018) Iterative co-creation for improved hand hygiene and aseptic techniques in the operating room: experiences from the safe hands study. *BMC Health Services Research* 18: 2.

Wikström E, Dellenborg L, Wallin L, et al. (2018) The Safe Hands Study: Implementing aseptic techniques in the operating room: Facilitating mechanisms for contextual negotiation and collective action. *American Journal of Infection Control*.
