# Supplementary material for: Effectiveness of implementing a preventive urinary catheter care bundle in hip fracture patients
Source: J Infect Prev. 2022 Feb 15;23(2):41–8. doi: 10.1177/17571774211060417 (PMC8941588; doi:10.1177/17571774211060417)
Supplement: sj-pdf-2-bji-10.1177_17571774211060417 – Supplemental Material for Effectiveness of implementing a preventive urinary catheter care bundle in hip fracture patients [file sj-pdf-2-bji-10.1177_17571774211060417.pdf]

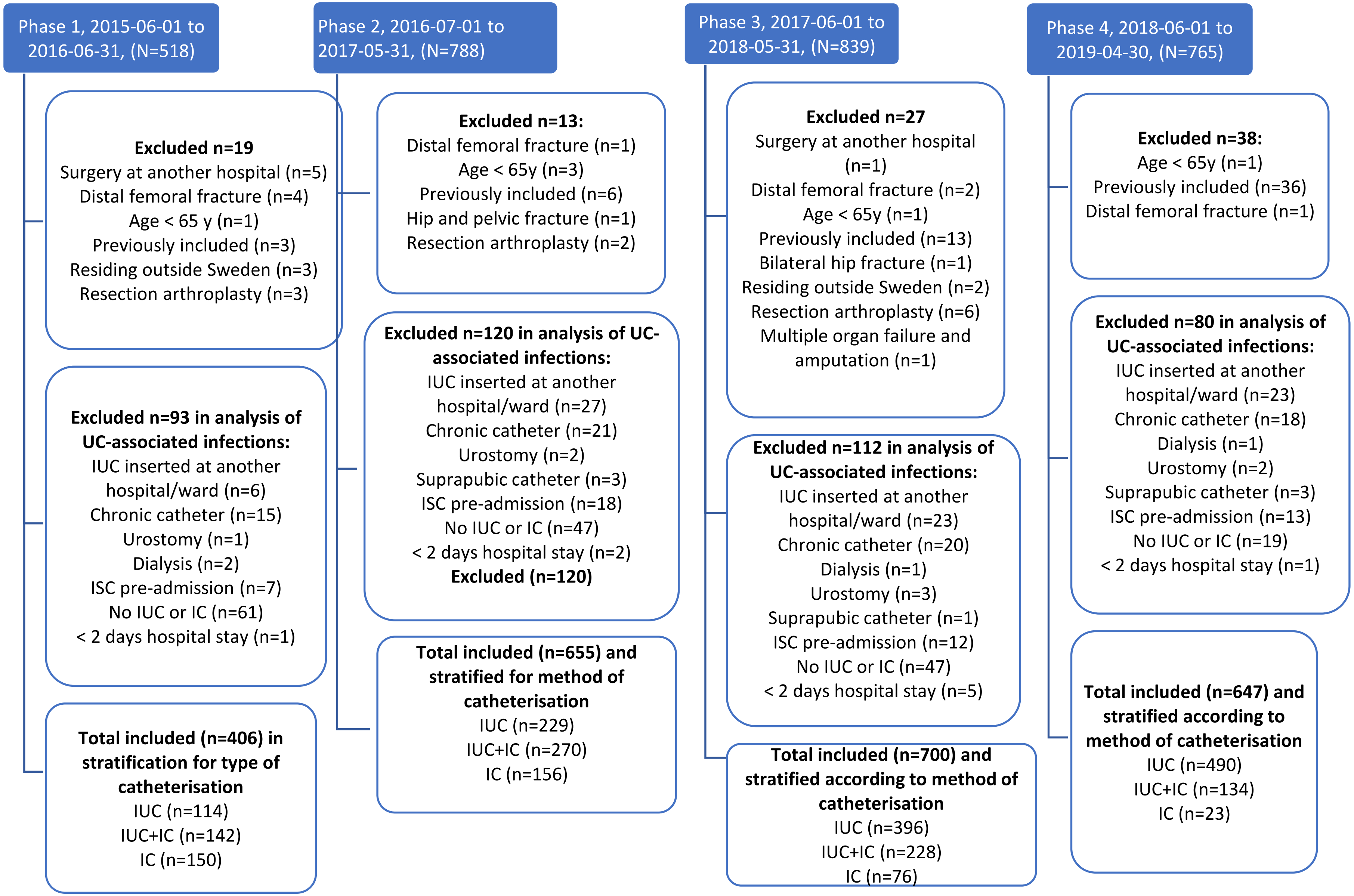

***Figure 1** Flowchart of included and excluded patients*

Abbreviations: urinary catheter = UC, indwelling urinary catheter = IUC, intermittent catheterisation = IC, intermittent self-catheterisation = ISC
