# Supplementary material for: Effectiveness of implementing a preventive urinary catheter care bundle in hip fracture patients
Source: J Infect Prev. 2022 Feb 15;23(2):41–8. doi: 10.1177/17571774211060417 (PMC8941588; doi:10.1177/17571774211060417)
Supplement: sj-pdf-3-bji-10.1177_17571774211060417 – Supplemental Material for Effectiveness of implementing a preventive urinary catheter care bundle in hip fracture patients [file sj-pdf-3-bji-10.1177_17571774211060417.pdf]

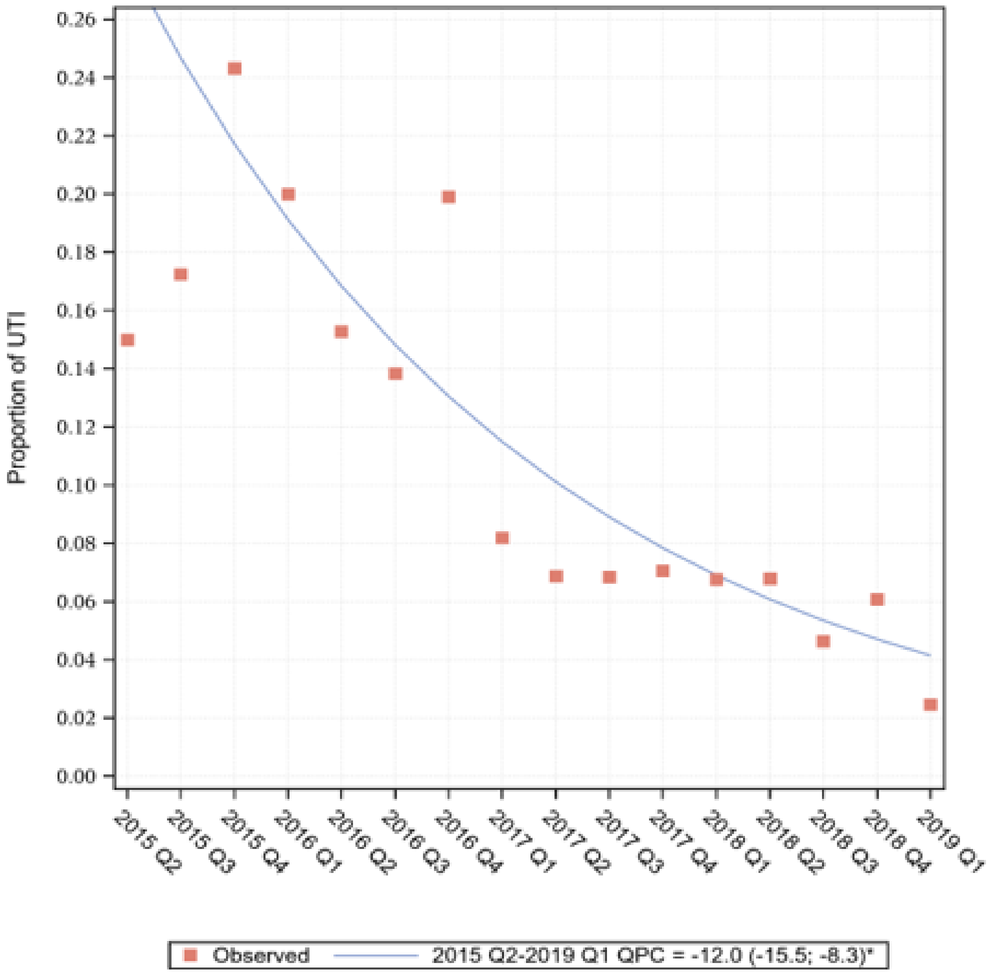

Figure 2. Jointpoint regression analysis. Indicates that the quarterly percentage change (QPC) is significantly different from zero, at the alpha level= 0.05, with 95% confidence intervals. Final selected model: 0 join points.
