# Supplementary material for: Effectiveness of implementing a preventive urinary catheter care bundle in hip fracture patients
Source: J Infect Prev. 2022 Feb 15;23(2):41–8. doi: 10.1177/17571774211060417 (PMC8941588; doi:10.1177/17571774211060417)
Supplement: sj-pdf-4-bji-10.1177_17571774211060417 – Supplemental Material for Effectiveness of implementing a preventive urinary catheter care bundle in hip fracture patients [file sj-pdf-4-bji-10.1177_17571774211060417.pdf]

Table 3. Location of indwelling catheter insertion, N=2,003, Phases 1-4

Location	**Phase 1**	**Phase 2**	**Phase 3**	**Phase 4**
	n=256	n=499	n=624*	n=624
Emergency room	41 (16.0)	85 (17.0)	122 (19.5)	211 (33.8)
Ortho-geriatric ward	61 (23.8)	90 (18.0)	174 (27.8)	198 (31.7)
Operating room	107 (41.7)	219 (43.8)	203 (32.5)	140 (22.4)
Post-operative ward	16 (0.06)	26 (0.05)	14 (0.02)	7 (0.01)
Inserted more than once	31 (12.1)	79 (15.8)	110 (17.6)	68 (10.8)

*n= numbers (%). *Missing value for one patient*
