# Supplementary material for: Effectiveness of implementing a preventive urinary catheter care bundle in hip fracture patients
Source: J Infect Prev. 2022 Feb 15;23(2):41–8. doi: 10.1177/17571774211060417 (PMC8941588; doi:10.1177/17571774211060417)
Supplement: sj-pdf-5-bji-10.1177_17571774211060417 – Supplemental Material for Effectiveness of implementing a preventive urinary catheter care bundle in hip fracture patients [file sj-pdf-5-bji-10.1177_17571774211060417.pdf]

Table 4. Univariable analyses of UC-associated infections for the patients with an acute hip fracture, N= 2 408

Variable		n (%)	OR* (95% CI)	*p*-value	Area under ROC curve (95%CI)
Phases (1-4)[1]	Phase 1	75 (18.5)	1.0		
	Phase 2	82 (12.5)	0.63 (0.45-0.89)		
	Phase 3	50 (7.1)	0.34 (0.23-0.50)		
	Phase 4	27 (4.2)	0.19 (0.12-0.30)	0.008	0.66 (0.62-0.69)
Age*	65-<82	66 (7.8%)			
	82-<89	78 (10.0%)			
	89-102	90 (11.5%)	1.02 (1.00-1.04)	0.015	0.55 (0.51-0.59)
Gender	Female	164 (9.6%)			
	Male	70 (9.9%)	1.03 (0.77-1.39)	0.83	0.50 (0.47-0.53)
ASA score*	I-II	94 (9.2%)			
	III-IV	140 (10.1%)	1.11 (0.84-1.46)	0.45	0.51 (0.48-0.55)
Diabetes	Yes	33 (9.0%)			
	No	201 (9.8%)	1.11 (0.75-1.63)	0.61	0.51 (0.48-0.53)
Hospital length of stay (days)*	3>6	7 (2.8%)			
	6<15	112 (7.7%)			
	15-68	115 (16.3%)	1.07 (1.05-1.08)	<0.0001	0.66 (0.62-0.70)
Catheterisation treatment	Indwelling urinary catheter	74 (6.0%)	0.41 (0.31-0.54)	<0.0001	0.61 (0.58-0.64)
	Indwelling urinary catheter + intermittent catheterisation	112 (14.5%)	2.10 (1.60-2.75)	<0.0001	0.59 (0.55-0.62)
	Intermittent catheterisation	48 (11.9%)	1.31 (0.94-1.84)	0.11	0.52 (0.49-0.55)
Urinary catheter days*	0.50-<2.50	66 (8.2%)			
	2.50-<5.00	45 (7.3%)			

	5.00-48.00	75 (12.8%)	1.07 (1.04-1.10)	<0.0001	0.57 (0.53-0.62)
Indwelling urinary catheter reinsertion	No	140 (8.2%)			
	Yes	46 (16.0%)	2.15 (1.50-3.08)	<0.0001	0.56 (0.53-0.59)
Intermittent catheterisation number of times*	0	74 (6.0%)			
	1-3	109 (11.6%)			
	4-5	26 (19.7%)			
	6-10	16 (17.6%)			
	11-15	6 (46.2%)			
	16-20	1 (50.0%)			
	21-25	2 (100.0%)	1.73 (1.51-1.98)	<0.0001	0.63 (0.59-0.67)

**Odds ratios were calculated on the underlying continuous variables.*

[1] *Phase 1 works as a reference analysis of phases 2-4.*
